# Is SARS-CoV-2 a Concern for Food Safety? A Very Low Prevalence from a Food Survey during the COVID-19 Pandemic in Northern Italy

**DOI:** 10.3390/foods11142096

**Published:** 2022-07-14

**Authors:** Sara Arnaboldi, Lucia Mangeri, Elisa Galuppini, Francesco Righi, Michela Tilola, Annalisa Scarazzato, Barbara Bertasi, Guido Finazzi, Giorgio Varisco, Virginia Filipello, Marina-Nadia Losio

**Affiliations:** 1 Food Safety Department, Istituto Zooprofilattico Sperimentale della Lombardia e dell’Emilia Romagna (IZSLER), Via A. Bianchi 9, 25124 Brescia, Italy; lucia.mangeri@izsler.it (L.M.); elisa.galuppini@izsler.it (E.G.); francesco.righi@izsler.it (F.R.); michela.tilola@izsler.it (M.T.); annalisa.scarazzato@izsler.it (A.S.); barbara.bertasi@izsler.it (B.B.); guido.finazzi@izsler.it (G.F.); giorgio.varisco@izsler.it (G.V.); virginia.filipello@izsler.it (V.F.); marinanadia.losio@izsler.it (M.-N.L.); 2 National Reference Centre for Emerging Risks in Food Safety (CRESA), Istituto Zooprofilattico Sperimentale della Lombardia e dell’Emilia Romagna (IZSLER), Via Celoria 12, 20133 Milan, Italy

**Keywords:** SARS-CoV-2, food safety, foods, mains water, viral detection, food monitoring

## Abstract

In 2019, SARS-CoV-2 was identified as the cause of an easily transmissible disease that was declared as a world pandemic. Foodborne transmission was never reported. However, early studies suggested that food could be involved in SARS-CoV-2 entry in the human gastrointestinal tract leading to possible infection, and highlighting the importance of further studies to inspect possible issues linked to food consumption. In this perspective, this work aimed at monitoring SARS-CoV-2 presence in some food and mains water samples in Northern Italy during the COVID-19 pandemic (2020–2022). A total of 1806 foods, 112 mains water samples, and 580 swabs on meat and dairy product surfaces were analyzed for SARS-CoV-2 RNA detection by Real-time PCR. All the analyzed samples were negative to viral RNA detection with the exception of one vegetable sample. Even if data on foodborne coronavirus transmission suggested a limited importance of this pathway, the impact of the current pandemic in Northern Italy deserved a rigorous investigation to rule out such possibility. Indeed, gaining insight on all SARS-CoV-2 possible transmission pathways, including the foodborne route, seemed of interest to maintain consumers’ confidence and trust in food safety, and for the effective management of the current, and future, possible pandemics.

## 1. Introduction

Coronaviruses are enveloped positive-sense RNA viruses belonging to the *Coronaviridae* family, that includes respiratory viruses infecting several animals with symptoms varying between different species [1,2,3]. In humans, coronavirus infection can lead to asymptomatic or mild-symptom infection (such as fever, cough, difficulty in breathing and gastrointestinal symptoms), or cause severe, even fatal, interstitial pneumonia [4,5]. Of the coronaviruses that are infective between humans, two species belonging to the *Betacoronavirus* genus were highly infectious and caused two outbreaks during the 21st century [6]. In particular, SARS-CoV is the etiological agent of Severe Acute Respiratory Syndrome (SARS) and caused an outbreak in 2002, mainly in China and Hong Kong, with a lethality of 9.6% [7,8]. In 2012, MERS-CoV was identified as being responsible for Middle East Respiratory Syndrome (MERS), and caused an outbreak in the Arabian Peninsula, with a lethality of 34% [9,10,11]. In 2019, a novel coronavirus (named SARS-CoV-2) was discovered, causing the Coronavirus disease 2019 (COVID-19), an easily transmissible disease with similar clinical characteristics to SARS and MERS [12]. Like other coronaviruses, SARS-CoV-2 has four structural proteins, known as the S (spike), E (envelope), M (membrane) proteins, that create the viral envelope, and the N (nucleocapsid) protein that contains the RNA genome. SARS-CoV-2 has a linear, positive-sense, and single-stranded RNA genome, approximately 30,000 bases long, sharing about 80% identity with that of SARS-CoV and about 96% with the bat coronavirus, suggesting bats were the possible source of SARS-CoV-2 [13,14]. In fact, MERS, SARS-CoV, and SARS-CoV-2 may have a zoonotic transmission, with the first SARS-CoV-2 infections linked to the Huanan Seafood Market (Wuhan, China) where various exotic live animals are sold [12,15,16,17]. SARS-CoV-2 spread rapidly worldwide, and COVID-19 was declared as a pandemic by the World Health Organization (WHO) on 11 March 2020 [18], involving 228 countries and territories around the world, with over 500 million confirmed cases, over 6 million deaths, and the lockdown of one-third of the world’s population [19,20,21]. To date, this pandemic represents a danger of extraordinary magnitude and exceptional impact for public health, also having serious repercussions on the global economy.

The main transmission route of SARS-CoV-2 is through respiratory droplets, mainly by direct contact with infected people [16,22,23,24,25]. Due to the emergency situation, close contact between people was mostly interrupted by restrictive measures in most of the affected countries, with the food sector being almost the only one not in lockdown [26]. In this situation, issues on the safety of food reaching consumers arise, despite the absence of evidence that the SARS-CoV-2, similar to SARS-CoV and MERS-CoV, foodborne transmission route plays an important role in the spread of the current outbreak, according to the WHO and the European Food Safety Authority (EFSA) [27,28,29]. In fact, coronaviruses cannot proliferate in foods as they need a living animal or human host to replicate [28], and the risk of foodborne infection can also be reduced by heating foods, as viruses are heat-sensitive. However, SARS-CoV-2 is stable at low temperatures, leading to possible transmission when fresh foods are exposed to droplets containing the virus before being frozen, suggesting a risk of SARS-CoV-2 transmission from food products that are part of the cold chain [30]. Finally, some recent studies hypothesized a possible SARS-CoV-2 fecal-oral transmission route, due to the viral isolation and RNA presence in fecal samples of infected patients (above all immune-compromised people) [31,32,33,34,35,36]. Gastrointestinal symptoms were reported during the previous SARS and MERS outbreaks [37,38], and also, in the COVID-19 pandemic, gastrointestinal manifestations were reported (in terms of loss of appetite, nausea, vomiting, diarrhea, and abdominal pain or discomfort) and were often associated with a more severe disease course [31,39]. Moreover, in a study by Cheung and colleagues it was reported that 70.3% of the studied patients had stools remaining positive to viral RNA detection, despite negative respiratory tests, suggesting that the viral gastrointestinal infection could persist even after viral absence in the respiratory tract, and highlighting the importance of gastrointestinal investigations for diagnostic and prognostic purposes [31,36,40]. Virus entry into intestinal epithelial cells appears to be mediated by the angiotensin converting enzyme 2 (ACE2) receptor and by a plasma membrane-associated type 2 transmembrane serine protease (TMPRSS2) [41,42]. Previous studies reported that cells expressing these receptors are the most affected by virus infection [43,44]. Recently, Lamers and colleagues studied the entry and replication of SARS-CoV-2 in human small intestinal organoids, demonstrating that the intestinal epithelium supports SARS-CoV-2 replication [45]. In fact, enterocytes from the small intestine and colonocytes actually co-express the entry genes (*ACE2* and *TMPRSS2*) in the lower gastrointestinal tract [46]. ACE2 receptors also modulate intestinal inflammation [47], with SARS-CoV-2 binding to ACE2 possibly resulting in gastrointestinal symptoms [42]. Actually, SARS-CoV-2 resists in a wide range of pH values (pH 3–10), but gastrointestinal secretions (pH 1.5–3.0) probably inactivate the virus, suggesting that, despite the gastrointestinal tract being susceptible to infection, SARS-CoV-2 further transmission is unlikely [42,48]. However, particular conditions (such as Hypochlorhydria) can lead to a pH increase, which can cause virus survival in intestinal cells, increasing the risk of enteric infections [49]. In addition, as can occur for other viruses, virions and RNA present in mucus can be protected, and not inactivated, by the acid gastrointestinal environment, allowing them to reach the intestine and, hence, their detection in feces [50].

To the best of our knowledge, literature about SARS-CoV-2 investigation in foods in Italy is lacking, despite the fact that studies are desirable to inspect possible issues linked to food consumption, both caused by food ingestion or contact with contaminated food. This work aimed at monitoring SARS-CoV-2 presence in the food categories of interest in investigations of the most common foodborne viruses (hepatitis A, hepatitis E, and norovirus), investigated by the routine analysis of the Food Safety Laboratory of the Istituto Zooprofilattico Sperimentale della Lombardia e dell’Emilia Romagna (IZSLER, Brescia) in Italy. This survey was performed during the pandemic in Northern Italy. Italy was one of the most severely affected countries in the world, with over 17 million cases diagnosed, and over 160 thousand deaths reported [20,51,52]. Therefore, considering the desired return to routine, the presence of SARS-CoV-2 in food is not negligible and must be better investigated, to maintain consumer confidence and trust in food safety.

## 2. Materials and Methods

### 2.1. Sampling

Samples were collected in Lombardy and the Emilia Romagna regions (Northern Italy), from January 2020 to April 2022, during the pandemic period. In these regions, there is a high population density and several food industries, with numerous COVID-19 cases reported. In particular, in the Lombardy region over 3.09 million cases were diagnosed with 40,800 deaths, whereas in the Emilia Romagna region over 1.59 million cases were diagnosed with 17,100 deaths since the start of the pandemic [20]. Samples were provided to the Food Safety Laboratory (IZSLER) by Competent Authorities (CAs) for official activities, or by Food Business Operators (FBOs) for their own check plans (Hazard Analysis and Critical Control Point, HACCP), for the analysis of viral contamination, according to ISO/TS 15216-2:2013 [53], and they were also analyzed for SARS-CoV-2 detection. The relevant food products comprised different food categories and matrices, including vegetables, fruit, berries, bivalve mollusks, fish products, meat products, processed food (gastronomic preparations/ready-to-eat foods—RTE), desserts, cheese, eggs, and pasta. Finally, mains water samples were also collected and analyzed in Lombardy and the Emilia Romagna regions (Northern Italy); in particular, the water analyzed was both well and ground water (intended for domestic use, for food washing, to be used in the industrial environment, and for irrigation or zootechnic use). In addition, swabs (FLOQSwabs™, COPAN ITALIA s.p.a., Brescia, Italy) from the surface of some meat and dairy products (hard and semi-hard cheeses) were collected, to test food surfaces for viral detection.

### 2.2. Sample Preparation, RNA Extraction and Purification

Food and water samples reached the Food Safety Laboratory (IZSLER) chilled (temperature of +5 °C ± 3 °C), and they were immediately analyzed. Food samples arrived in the package intended for sale that was removed before the weighing phase. The sample preparation was different, based on the sample matrix, according to ISO/TS 15216-2:2013 [53], or to an internal preparation protocol for those matrices not included in the ISO procedure.

#### 2.2.1. Bivalve Mollusks

A minimum of 2 g of hepatopancreas was collected and coarsely homogenized, 10 µL of mengovirus (recombinant mengovirus-vMC_0_ strain, ATCC VR-1597™; 10^4^ viral particle/μL) were spiked into every sample as process control, as per the ISO/TS 15216 2:2013 [53], and 2 mL of proteinase K solution were added to the homogenate sample. Samples were incubated at 37 °C for 1 h, and then at 60 °C for 15 min. After centrifugation at 3000× *g* for 5 min the eluate was recovered for viral RNA extraction.

#### 2.2.2. Vegetables, Fruit, and Berries

Briefly, 25 g of the edible sample surface were finely cut and washed with TGBE buffer (pH 9.5), and 10 μL of mengovirus were spiked into each sample. For berries and fruit, pectinase was added. The eluate was concentrated with 5× PEG/NaCl solution and, only for berries, a further chloroform/butanol purification step was performed.

#### 2.2.3. Mains Water

Each sample (500 mL) was spiked with 10 µL of mengovirus and filtered; to wash the membrane, 3 mL of beef extract (3%, pH 9.5) were added. After incubation at room temperature for 5 min, the eluate was used for viral RNA extraction.

#### 2.2.4. Processed Food (Gastronomic Preparations/RTE Foods, Crustaceans, Fish Products, Desserts, Cheese, Eggs, Pasta)

Viral concentration was performed following an internal preparation method. A total of 50 g of sample were spiked with mengovirus and 1:1 (*w*/*v*) glycine buffer (50 mM, pH 9.2). When the sample was multicomponent, the 50 g had to include all the matrices contained in the product. The sample was then spun at 10,000× *g* for 20 min at 4 °C; the supernatant was added with 1:4 (*v*/*v*) PEG_8000_ solution, and kept at 4 °C overnight for precipitation. The day after, a centrifugation at 10,000× *g* for 45 min at 4 °C was performed, the supernatant was discarded, and the pellet was resuspended with 10 mL of sterile water. After another centrifugation at 10,000× *g* for 20 min at 4 °C, 1:4 (*v*/*v*) PEG_8000_ solution was added to the supernatant, mixed, and left overnight at 4 °C for a second precipitation. The third day, after a centrifugation at 10,000× *g* for 45 min at 4 °C, the supernatant was discarded, and the pellet was resuspended with 1 mL of sterile water. After another centrifugation at 10,000× *g* for 20 min at 4 °C, the supernatant was recovered for RNA extraction.

#### 2.2.5. Meat Products

For meat sample preparation, 50 g of tissue were finely cut and 5 g of sample were put into a sterile bag with filter, and 7 mL of QIAzol Lysis Reagent (Qiagen, Hilden, Germany) were added. Then, 10 µL of mengovirus were spiked into every sample. The samples were homogenized with the TissueLyser (Qiagen, Hilden, Germany) at maximum speed for 2 min. The liquid was recovered and centrifuged at 10,000× *g* for 45 min at 4 °C. The supernatant was recovered and 1.4 mL of Chloroform (1 M) was added. Samples were left at room temperature for 15 min, and after centrifugation at 10,000× *g* for 15 min at 4 °C, the supernatant was ready for RNA extraction.

#### 2.2.6. Food Swabs

Nylon flocked swabs (FLOQSwabs™, COPAN ITALIA s.p.a., Brescia, Italy) were used to investigate viral presence on meat and dairy product surfaces. For each sample, an area of 10 cm^2^ was swabbed, then the swab was immediately transferred in a dedicated transport medium (eNAT^®^, COPAN ITALIA s.p.a., Brescia, Italy) formulated to ensure viral inactivation and nucleic acid preservation. A total of 10 μL of mengovirus were spiked into every eNAT^®^ tube (eNAT^®^, COPAN ITALIA s.p.a., Brescia, Italy), and 500 μL of the liquid were used for viral RNA extraction.

#### 2.2.7. RNA Extraction and Purification

Viral RNA was extracted and purified using the NucliSENS^®^ MiniMag kit (bioMérieux SA, Marcy-l’Etoile, France) according to the manufacturer’s instructions, as suggested by ISO/TS 15216-2:2013 [53]. A negative extraction control was processed with every run of extraction. The eluted RNA was stored at −80 °C until use.

### 2.3. SARS-CoV-2 One-Step RT Real-Time PCR

SARS-CoV-2 RNA detection was performed by One-step RT Real-time PCR targeting the *ORF1ab* region (coding for non-structural protein 14), *Sarbeco E* gene (coding for the envelope protein), and the *N1* and *N3* portions of the gene *N*, coding for the nucleoprotein. SARS-CoV-2 was detected using specific primers and TaqMan probes described by La Rosa et al., 2021 (for *ORF1ab* region) [52], Corman et al., 2020 (for *Sarbeco E* gene) [54], and suggested by the Centers for Disease Control and Prevention (for *N1* and *N3* regions) [55], that are shown in Table 1. A sample was considered positive when at least one of the four targets resulted in positive reading in the RT Real-time PCR test.

Samples were analyzed in four different reactions (one for each target gene detection). In particular, to detect the *ORF1ab* region, *N1* gene, and *N3* gene, three separate reactions were performed using the RNA UltraSense™ One-Step Quantitative RT-PCR System (Invitrogen, Carlsbad, CA, USA). A total volume of 25 μL contained 5 μL of Ultrasense reaction mix (5×), 1 μL of each primer (12.5 μM and 22.5 μM, Forward and Reverse, respectively), 1 μL of probe (6.25 μM), 0.5 μL of Rox reference dye (50×), 1.25 μL of RNA Ultrasense enzyme mix, 10.25 μL of DNAse-RNase-free water (Sigma–Aldrich, St. Louis, MO, USA), and 5 μL of extracted RNA. All the reactions were performed in a CFX96 Touch™ Real-Time PCR Detection System (Bio-Rad, Hercules, CA, USA) with the following thermal profiles: (i) for *ORF1ab* detection: reverse transcription at 50 °C for 30 min, denaturation at 95 °C for 10 min, and 45 amplification cycles at 95 °C for 15 s, annealing/extension at 60 °C for 45 s; (ii) for *N1* and *N3* genes detection, the reverse transcription was performed at 45 °C for 30 min, then denaturation at 95 °C for 10 min, and 45 amplification cycles at 95 °C for 10 s, annealing/extension at 55 °C for 30 s. Positivity was detected when Ct ≤ 41.

The reaction used to detect the *Sarbeco E* gene was performed using the SuperScript™ III One-Step RT-PCR System with Platinum™ Taq DNA Polymerase (Invitrogen, Carlsbad, CA, USA). A total volume of 25 μL contained 12.5 μL of 2× Reaction mix (1×), 1 μL of each primer (400 nM), 0.5 μL of probe (200 nM), 1 μL of SuperScript™ III RT/Platinum™ Taq mix, 4 μL of DNAse-RNase-free water (Sigma-Aldrich, St. Louis, MO, USA), and 5 μL of extracted RNA. The reaction was performed in a CFX96 Touch™ Real-Time PCR Detection System (Bio-Rad, Hercules, CA, USA) with the following thermal profile: reverse transcription at 52 °C for 15 min, denaturation at 94 °C for 2 min, and 45 amplification cycles at 94 °C for 15 s, annealing/extension at 60 °C for 30 s. Positivity was detected when Ct ≤ 39.44.

Negative and positive amplification controls were included in each run for each target detection.

### 2.4. Mengovirus One-Step RT Real-Time PCR

Mengovirus detection was used to validate the process; samples with negative mengovirus amplification needed to be repeated from RNA extraction.

RT Real-time PCR was performed using primers and TaqMan probe shown in Table 2, to confirm the process effectiveness.

The reaction was performed using RNA UltraSense™ One-Step Quantitative RT-PCR System (Invitrogen, Carlsbad, CA, USA) in a total volume of 20 μL containing 5 μL of Ultrasense reaction mix (5×), 1 μL of each primer (12.5 μM and 22.5 μM, Forward and Reverse, respectively), 1 μL of probe (6.25 μM), 0.5 μL of Rox reference dye (50×), 1.25 μL of RNA Ultrasense enzyme mix and 10.25 μL of DNAse-RNase-free water (Sigma–Aldrich, St. Louis, MO, USA). Five μL of RNA template were added to the reaction mix, and positivity was detected when Ct ≤ 40.

The reaction was performed in a CFX96 Touch™ Real-Time PCR Detection System (Bio-Rad, Hercules, CA, USA). Reverse transcription was performed for 1 h at 55 °C; samples were then incubated at 95 °C for 5 min and amplified for 45 cycles of 15 s at 95 °C, 1 min at 60 °C and 1 min at 65 °C. Negative and positive amplification controls were included in each run.

### 2.5. Data Analysis

Prevalence was calculated as the ratio between positive samples and total samples and was expressed as a percentage. The prevalence and 95% confidence interval (95% CI) were calculated by Wilson’s method using Epitools software [56].

## 3. Results

A total of 2498 samples were collected in Northern Italy from January 2020 to April 2022. In particular, a total of 1806 food samples were collected (819 in 2020, 853 in 2021, and 134 in 2022). In addition, a total of 112 samples of mains water were collected and analyzed (65 samples in 2020, and 47 samples in 2021). Finally, a total of 580 swabs (300 on meat products, and 280 on dairy products) were collected to test food surfaces for viral detection. Concerning swabs on meat products, a total of 210 and 90 samples were collected in 2021 and 2022, respectively, whereas concerning those of dairy products, a total of 210 and 70 swabs were collected in 2021 and 2022, respectively. The different food samples collected and analyzed are described in Table 3.

All the collected samples were analyzed for SARS-CoV-2 RNA detection by RT Real-time PCR targeting the *ORF1ab* region, *Sarbeco E* gene, and the *N1* and *N3* portions of the *N* gene. All the analyzed food samples were negative for *ORF1ab*, *N1*, and *N3* genes detection. Only one vegetable sample (green salad) was positive to *Sarbeco E* gene detection, with a prevalence of 0.06% (95% CI 0.01–0.31%), including all food samples analyzed (1806 samples), and of 0.21% (95% CI 0.04–1.16%), considering the vegetable samples (486 samples). Finally, focusing on the salad samples analyzed (352 samples), a prevalence of 0.28% (95% CI 0.05–1.59%) was obtained.

None of the mains water samples or swabs on meat and dairy product surfaces were positive to any of the four genes investigated.

Mengovirus was detected in each sample, making the results reliable.

## 4. Discussion

The food industry has food safety management systems based on HACCP principles to manage food safety risks, and prevent food contamination, including by viral foodborne pathogens [29]. Effective food inspections are indeed essential to control viruses’ spread along the food chain, and implementing the monitoring of SARS-CoV-2 in foods might also be of interest to increase consumers’ trust, by ensuring the safety of the food products reaching their tables [57]. In fact, recent studies reported a concrete risk of SARS-CoV-2 replication in the gastrointestinal tract, leading to gastrointestinal symptoms, often aggravating the course of COVID-19 infection [31,42,45,49], despite SARS-CoV-2 foodborne infection or transmission having never been demonstrated [27,28,58]. In this perspective, investigating the presence of SARS-CoV-2 in the food sector appeared of interest to better understand other possible alternatives in SARS-CoV-2 transmission pathways, and to provide insight on possible food-safety-related risks.

In the current study, the monitoring of foods was performed during the pandemic (2020–2022), in an area (Northern Italy) were SARS-CoV-2 was widespread, with lots of confirmed infections and deaths [51,52,59]. The food categories analyzed were chosen as they were considered at possible risk of contaminations by viruses frequently causing enteric illnesses in humans (hepatitis A, hepatitis E, and norovirus). In particular, foods commonly consumed raw or lightly cooked (such as berries, fresh fruit, and vegetables) were analyzed, as they are considered at risk of transmitting enteric viruses, also because they undergo large human handling [60,61,62,63]. Indeed, in these products, viral contamination (which also possibly applies to SARS-CoV-2) can occur during human harvesting, processing, packaging, or distribution (through handling by infected workers), and also through environmental contamination pre-harvest and post-harvest, by contaminated water for irrigation or washing [64]. Accordingly, it seemed important to investigate viral presence in the mains water used for irrigation, washing of fresh fruit/vegetables and RTE foods (both in domestic or industrial situations), or for human/animal drinking. Other food products at high risk of viral contamination that were analyzed were mollusks, crustaceans, and fish products grown in waters with possible fecal or sewage contamination (considering that SARS-CoV-2 was detected in feces and sewage [35,65,66,67]). Concerning bivalve mollusks, the virus possibly being present in seawater would be concentrated in the hepatopancreas through filtration, as commonly occurs for enteric viruses, increasing the risk of ingestion of high viral concentrations in raw products [68,69]. The other food categories analyzed were food products mainly at risk of secondary contamination, possibly due to contact with contaminated materials used for processing or packaging. In fact, recent studies reported SARS-CoV-2 stability on different materials and at low temperatures, suggesting a risk of SARS-CoV-2 persistence in food products that are distributed within a cold chain, such as fish or meat products [30,48,70].

SARS-CoV-2 detection with Real-time PCR in food samples resulted in the identification of only one green salad positive to *Sarbeco E* gene (with a prevalence of 0.06%, 95% CI 0.01–0.31%), whereas all samples were negative for *ORF1ab*, *N1* and *N3* genes detection. Due to the very low prevalence found in the vegetable samples analyzed (0.21%, 95% CI 0.04–1.16%), and in salad samples too (0.28%, 95% CI 0.05–1.59%), the main hypothesis was that SARS-CoV-2 RNA presence on the salad was triggered by a secondary contamination, possibly due to contact with an infected worker or with a contaminated surface or tool in the production step. However, the contamination evidence was based on RNA detection. There is still little information linking the presence of viral genomes in foods to virus infectivity, and viral isolation in cell cultures is needed to assess if the detected RNA represented a viable virus able to infect [71,72]. SARS-CoV-2 is not considered a foodborne virus [73], and the very low prevalence data of its RNA detection in food products was certainly reassuring and useful to maintain customer trust, demonstrating that the following of strict hygiene and control procedures could minimize the risk of virus contamination from foods.

Despite the emergency situation, significant efforts have been made to rapidly develop tools for SARS-CoV-2 detection in foods, which have relatively challenging matrices for accurate viral accurate. Indeed, unlike bacteria, viral quantity cannot be easily increased with specific enrichment protocols, so detection methods must be sensitive and specific [74]. At present, one of the best approaches for SARS-CoV-2 detection which also applies to food matrices, is the detecting of RNA by RT Real- time PCR, a highly specific and sensitive method [75]. In this study a process control (mengovirus) was added in each sample to check possible food-matrix inhibition, and viral concentration was performed before RNA extraction, to ensure successful virus detection. Moreover, only the external surface or peel was analyzed, as coronaviruses can replicate only in living human or animal hosts [28], so they cannot spread into foods, remaining on their surfaces.

Finally, the sampling of food products was not completely representative of the different investigated food categories. In fact, no specific collection scheme was established by the authors (as part of a not-collecting Laboratory) because samples were provided by CAs and FBOs within their own collection plans. In particular, collection plans, their frequency, and sample size were determined by CAs and FBOs according to the risks and criteria for the risk categorization [76,77]. However, an extensive number of samples were processed during the pandemic period in an area with a high number of COVID-19 cases [20], with an extensive coverage and diversity of food products, improving data on SARS-CoV-2 in foods, and helping to decrease consumers’ concerns regarding possible transmission through foods.

In addition, a total of 580 swabs were sampled from the surface of meat and dairy products. Indeed, the nylon flocked swabs used for sampling were a valid tool for viral detection on food surfaces, improving sample absorption, and allowing the release of a high quantity of viruses possibly present on the sample surface. All the analyzed surface swabs were negative for SARS-CoV-2 detection, suggesting that SARS-CoV-2 contamination is unlikely in these food categories, despite processed foods, having extensive handling procedures, possibly leading to secondary contamination. However, any secondary viral contamination of food stuffs is minimized by following hygiene and sanitation rules, and safety procedures from the initial supply of the raw material (i.e., harvesting for vegetables, or slaughter for fish/meat products), to processing, packaging, and distribution, recommended also by WHO and EFSA [27,29,78]. A recent study in a retail store suggested preventive measures and sanitizing routines were necessary methods to minimize the exposure risk from contaminated high-touch surfaces [79,80]. Literature reported that the greatest risk for the food industry is transmission among workers, further highlighting the need for innovative and effective disinfection strategies that must be implemented in the workplace to avoid SARS-CoV-2 transmission [81,82]. Finally, even when an infected person transfers the virus on a food surface or packaging (by direct sneezing or coughing, or through contaminated hand-touch), the viral transmission is possible only for a short time after contamination (by self-touching the mucous membranes of nose or eyes), because the viral load is expected to decrease over time, as viruses cannot proliferate in food [83]. Nevertheless, viruses in food are possibly at lower concentration compared to respiratory droplets, so the infection likelihood is lower than through the latter [84].

Concerning mains water, the negativity of all the analyzed samples was reassuring, suggesting that the water used for irrigation, food processing, washing, or even drinking in the analyzed area is SARS-CoV-2-free, probably thanks to effective disinfection procedures, although the virus survival in water is estimated up to six days [85]. These results were in accordance with a recent study that detected SARS-CoV-2 RNA in a river in Northern Italy with negligible vitality, indicating the absence of sanitary and environmental risk of infection from surficial water [86]. However, until the pandemic is declared over, continuous monitoring of water used both for irrigation and human use is crucial to ensure the protection of human health, and the safety of the production processes.

The purpose of this study was a survey on SARS-CoV-2 presence in foods and mains water in a globally difficult period, in which concern about food safety has increased. This work suggested a negligible risk of SARS-CoV-2 contamination associated with foods and mains water, assuaging concerns regarding food products as possible SARS-CoV-2 transmission vehicles. In this period, these data are reassuring and could further support recovery from the impacts of COVID-19.

## 5. Conclusions

Considering the extreme impact of this pandemic on all aspects of human life, it should be underlined that food production and supply must be considered a non-negligible sector; indeed, food is indispensable and cannot be locked down. The safety of food and the environment in which food is produced, processed, and delivered must therefore be guaranteed, through careful and constant compliance with hygiene rules and protocols [26,57]. Indeed, the management of a pandemic cannot only concern the screening of populations, but also the monitoring of food, and processing surfaces and environments [57].

In conclusion, the data of the current study suggested that SARS-CoV-2 infection, or its spread through the consumption of, or contact with, contaminated foods is of minor concern for the current pandemic in Northern Italy, and contributed to ruling out the possibility of foodborne transmission.

## Figures and Tables

**Table 1:** Primers and probes used to detect SARS-CoV-2 in One-Step RT Real-time PCR.

Region	Name	Type	Sequence	Reference
*ORF1ab*	CoV2 F	Forward	5′-ACATGGCTTTGAGTTGACATCT-3′	
	CoV2 R	Reverse	5′-AGCAGTGGAAAAGCATGTGG-3′	La Rosa et al., 2021
	CoV-2-P	Probe	5′-FAM-CATAGACAACAGGTGCGCTC-MGBEQ-3′	
*Sarbeco E*	E_Sarbeco_F1	Forward	5′-ACAGGTACGTTAATAGTTAATAGCGT-3′	
	E_Sarbeco_R2	Reverse	5′-ATATTGCAGCAGTACGCACACA-3′	Corman et al., 2020
	E_Sarbeco_P1	Probe	5′-FAM-ACACTAGCCATCCTTACTGCGCTTCG-BHQ1-3′	
*N1*	N1 F	Forward	5′-GACCCCAAAATCAGCGAAAT-3′	Centers for Disease Control and Prevention
	N1 R	Reverse	5′-TCTGGTTACTGCCAGTTGAATCTG-3′	
	N1 P	Probe	5′-FAM-ACCCCGCATTACGTTTGGTGGACC-BHQ1-3′	
*N3*	N3 F	Forward	5′-GGGAGCCTTGAATACACCAAAA-3′	Centers for Disease Control and Prevention
	N3 R	Reverse	5′-TGTAGCACGATTGCAGCATTG-3′	
	N3 P	Probe	5′-FAM-AYCACATTGGCACCCGCAATCCTG-BHQ1-3′	

**Table 2:** Primers and probe used to detect mengovirus in One-Step RT Real-time PCR.

Name	Type	Sequence
Mengo 110	Forward	5′-GCGGGTCCTGCCGAAAGT-3′
Mengo 209	Reverse	5′-GAAGTAACATATAGACAGACGCACAC-3′
Mengo 147	Probe	5′-FAM-ATCACATTACTGGCCGAAGC-MGB-3′

**Table 3:** Number and categories of food, mains water, and swab samples collected in Northern Italy and analyzed from 2020 to 2022 to detect SARS-CoV-2 RNA. In brackets the details of the products included in the sampling are reported.

Food Categories	Number of Samples
Bivalve molluscs (oyster, mussel, clam, razor clam)	759
Vegetables (carrot, salad, tomato, spinach)	486
Berries	320
Fruit (pineapple, apple, cherry, kiwi)	123
Gastronomic preparations/Ready-to-eat foods	64
Meat products (bovine, swine)	30
Crustaceans (lobster, shrimp)	11
Fish products (trout, cod, salmon, sardine, octopus)	6
Desserts	4
Cheese	1
Eggs	1
Pasta	1
**Total Food Samples**	**1806**
Mains water samples	112
Swabs on meat products	300
Swabs on dairy products (hard or semi-hard cheese)	280
**Total Samples**	**2498**

## Data Availability

Data is contained within the article.
